# Quality of energy intake in Malaysian adolescents: prevalence, characteristics, determinants and impact of implausible reporters

**DOI:** 10.1017/S1368980022000726

**Published:** 2022-03-24

**Authors:** Mohamed S Zulfarina, Razinah Sharif, Ahmad Mohd Sharkawi, Sabarul Afian Mokhtar, Ahmad Nazrun Shuid, Isa Naina-Mohamed

**Affiliations:** 1 Pharmacoepidemiology Unit, Department of Pharmacology, Faculty of Medicine, Universiti Kebangsaan Malaysia Medical Centre, Jalan Yaacob Latif, Bandar Tun Razak, Cheras, Kuala Lumpur 56000, Malaysia; 2 Nutritional Science Program and Centre for Healthy Ageing and Wellness, Faculty of Health Sciences, Universiti Kebangsaan Malaysia, Jalan Raja Muda Abd Aziz, Kuala Lumpur, Malaysia; 3 ProVice-Chancellor Office, Health Campus, Jalan Yaacob Latif, Bandar Tun Razak, Cheras, Kuala Lumpur, Malaysia; 4 Department of Pharmacology, Faculty of Medicine, Universiti Teknologi MARA, Sungai Buloh Campus, Jalan Hospital, Sungai Buloh, Malaysia

**Keywords:** Misreport, FFQ, Intake estimates, Overweight, Obese

## Abstract

**Objective:**

Misreporting of energy intake (EI) in nutritional epidemiology is common and even severe among adolescents. Thus, the current study aims to examine the presence, bias and impact introduced by implausible reporters.

**Design:**

Cross-sectional.

**Setting:**

Central and eastern regions of Peninsular Malaysia.

**Participants:**

A stratified random sampling was employed to select 917 secondary school-going adolescents (aged 15–17 years).

**Results:**

The prevalence of under-reporters was 17·4 %, while no over-reporters were identified. Under-reporters had higher body composition and lower dietary intakes (except for vitamin C, Cr and Fl) compared with plausible reporters (*P* < 0·05). Adolescents with overweight and obesity had a higher odds of under-reporting compared with under-/normal weight adolescents (*P* < 0·001). In model 3, the highest regression coefficient (*R* ^2^ = 0·404, *P* < 0·001) was obtained after adjusting for reporting status.

**Conclusions:**

Overweight and obese adolescents were more likely to under-report their food intake and consequently affect nutrient intakes estimates. Future analyses that include nutrient intake data should adjust for reporting status so that the impact of misreporting on study outcomes can be conceded and consequently improve the accuracy of dietary-related results.

Accurate valuation of dietary intake is a prerequisite in a nutritional epidemiology study. Acquiring dietary reporting that could accurately represent true dietary consumption (usual intake) is undeniably difficult due to cognitive abilities and dietary habits^(1)^. Nutritional study often relies on subjective dietary intake due to the feasibility issue of questionnaires, notably in large-scale studies^(2)^. Inevitably, data from self-reported dietary intakes tend to be misreported, which include both under-report and over-report. Under-reporting is particularly prevalent among adolescents; it varies between ten and 50 %^(3)^ and appears to happen both randomly and systematically^(4)^.

The extent of misreporting depends on several contributing factors, including instruments for dietary assessment, the method and cut-off used to identify implausible reporters^(5)^. Error while reporting also happened when a person is selective to certain kinds of foods that are likely to be socially undesirable such as high fat, added sugars and alcohol, which is also known as selective misreporting^(4–6)^ or sudden change in eating behaviour, which happens if a person eats less or more than usual (under/over-eating)^(7)^ or could also be linked with a person’s level of adiposity or body weight status. This occurred when respondents with certain characteristics, for example, overweight and obese persons, under-estimate (under-record) the amounts of food consumed compared with their counterparts and vice-versa, also referred to as differential misreporting^(4–6,8)^. Nevertheless, study on bias reports of energy intake (EI) served as a surrogate assessment for investigating the discrepancy between self-reported dietary intake and actual dietary consumption^(1)^.

Energy consumption is the basis of diet, as all nutrients must be provided within the amount of food required to meet the energy requirement. Thus, if the amount of EI is underestimated, it is plausible that consumption of other nutrients is also underestimated^(9)^. Currently, one of the widely used methods to determine invalid reporting of EI is the assessment of EI against presumed energy requirements. The reported EI which was estimated from equations expressed as multiples of the mean basal metabolic rate (BMR) was then compared with the presumed mean energy expenditure of the studied population, expressed as multiples of BMR^(9–11)^. This method that was first developed by Goldberg *et al*.^(12)^ and later revised by Black^(7,10)^ has been employed in several studies conducted among children and adolescents^(4,5,8,13,14)^.

Implausible reporting affects the estimation of EI and consequently of other nutrients. Data sets that included misreporting could reduce the accuracy of reported EI by increasing the risk of type 2 error (bias towards the null). In some instances, inaccurate reporting may diminish the usefulness of nutrition data by having the possibility to attenuate the association between food/nutrient intake and health/disease outcomes. Besides, the consequent probability for differential errors in dietary data may obfuscate the interpretation of diet–disease relationships and at worst could yield ambiguous links between diet and disease^(1)^.

Thus far, there are no clear guidelines on handling misreporting, with only a limited number of studies that examined the statistical method of handling implausible EI in children^(15)^ and adolescents^(3)^. Moreover, study of misreporting among children and adolescents^(4,8,13,14,16)^, particularly in Asia, is sparse^(5,6)^. Furthermore, differences in dietary habits due to the heterogeneity of the study population may hinder the extrapolation of findings from other countries to heterogenic countries such as Malaysia and its neighbouring countries. This warrants an exclusive investigation that can be truly representative of the study population. Thus, the current study aims (1) to identify the prevalence of implausible reporters using a method based on the ratio of EI to BMR; (2) to identify the characteristics and lifestyle determinants associated with implausible reporters and (3) to compare the viability of statistical methods to correct misreporting of EI in a varied socio-demographic population of Malaysian adolescents.

## Methods

This is a cross-sectional study conducted among the public secondary school adolescents (aged 15–17 years) in the central and eastern regions of Peninsular/West Malaysia: Selangor (central part), Federal Territory of Kuala Lumpur (metropolitan area) and Pahang (eastern region) state. Data collection was conducted between September 2014 and July 2015.

### Sampling

Stratified random sampling was employed to select schools and participants. In summary, public schools were randomly selected to represent different niches based on the national school system, ethnicity and location (daily school-urban, daily school-rural, daily-vernacular, boarding, sports school and Orang Asli (Indigenous) dominated school)^(17)^. All fourth-year students in the selected secondary schools were invited to participate. For the purpose of this current study, however, only students who returned the agreed written consent forms (for participants and their parents or carers) and had no concurrent medical conditions were recruited into the study. All valuations were collated at the recruitment sites by a trained enumerator. In summary, 1078 adolescents from six public secondary schools were invited. A total of 960 adolescents returned with consented form; however, only 917 participants fulfilled the eligibility criteria to be included in the final analysis; twenty-two adolescents were not in the age range, fifteen adolescents were non-resident of Peninsular Malaysia (Indigenous of Borneo Island) or of other ethnicity and six adolescents had obscured FFQ answers.

### Measurements

Dietary supplement intake was extracted using a validated dietary supplement questionnaire for adolescents^(18)^. Demographic information (gender at birth, age, ethnicity and school types), smoking and alcohol intake status were obtained as part of the questionnaire. Eligible adolescents were grouped to Malay, Chinese, Indian or Orang Asli for ethnicity, while urban, rural, boarding or sports school for types of school^(17)^. Participants were identified as either occasional (defined as drinking at most once a month) or non-drinkers (defined as never, tried once or two)^(17)^. While smoking status was categorised as smokers (at least one puffs a day, sometimes) or non-smokers (tried once and never)^(17)^.

### Anthropometric and body composition

Height was measured using a portable stadiometer to the nearest 0·1 cm (model 213 SECA). Body weight was measured in light clothing on a portable body composition analyser (BC-420 MA Tanita) that integrates a bioelectrical impedance analysis, a validated technique that was used to estimate components of body composition including lean mass and fat percentage^(19)^. All measurements were performed in triplicate and followed the standard procedure^(20)^. The age- and sex-specific BMI *Z*-score (BMI-*Z*) was calculated using the least mean square method^(21)^. Weight status was defined using the new and extended International Obesity Task Force cut-offs reference^(21)^. The new age-sex specific BMI for children and adolescents aged two to 18 years, cut-offs must be on the BMI percentile corresponding at age 18 years, through the values less than 18·5 kg/m^2^, more than 25 kg/m^2^ and 30 kg/m^2^ for underweight, overweight and obese, respectively.

### Physical activity and sedentary behaviour

The individual physical activity level (PAL) was determined using a validated Malay short version of the International Physical Activity Questionnaire^(22)^. The frequency (number of d/week) and duration (min/d) of physical activity (walking, moderate-intensity activities and vigorous-intensity activities) undertaken during the previous 7 d was assessed. Energy expenditure in terms of multiples RMR or MET-minutes per week (MET-min/week) was computed for each type of activity. The total energy expenditure was calculated as the sum of vigorous activities, moderate activities and walking MET-min/week. Adolescents who performed vigorous intensity activity on at least 3 d achieving a minimum total score of 1500 MET-min/week or combination of vigorous PA, moderate PA and/or walking for at least 7 d achieving a minimum total score of 3000 MET-min/week were classified as high PA. Adolescents were categorised as moderate PA if they performed vigorous PA for at least 20 min/d for at least 3 d, moderate PA for at least 30 min/d for at least 5 d or a combination of vigorous PA, moderate PA and/or walking for at least 5 d, achieving a total score of at least 600 MET-min/week. Conversely, adolescents who did not achieve any criteria listed above were grouped as low physical activity^(22)^.

Participants were also asked to estimate the total time (min/d) of sitting or lying down during the weekdays for the past 7 d. Adolescents were asked to consider the time while at school, at home, while doing coursework and leisure time, including time spent sitting at desk, visiting friends, reading, sitting or lying down in front of screen. Total daily sitting time was used as a proxy of sedentary behaviour. Adolescents were categorised as high sedentary when the total sitting time was at least 8 h/d^(23)^.

### Dietary intakes assessment

Dietary habit was determined from a validated FFQ, designed explicitly for the local population and has a moderate average Pearson correlation of *r* = 0·416^(24)^. Adolescents were required to report the frequency of consumption for each of the ninety-four selected foods and beverages commonly consumed during the preceding month and the quantities/amount consumed at each occurrence. Examples of portion size estimated using local household measures were given to facilitate answering the questions. Each FFQ was reviewed for completeness and accuracy of the information. Any omission or ambiguities were clarified with participants via direct interview. The data obtained were converted into kilocalorie and nutrient intakes based on the Nutrient Composition of Malaysian Foods^(25)^ and Atlas of Food Exchanges & Portion Size^(26)^ using the Nutritionist Pro™ Diet Analysis version 4 (Axxya Systems, Stafford, Texas). This present study includes nutrients that are presented in the latest and updated Malaysia Recommended Nutrient Intakes, which is the main reference for national nutrient intake^(27)^.

### Relative energy and nutrient intake

The Goldberg cut-offs or confidence limit of agreement was applied to determine invalid reporting of EI^(12)^. The formula compares EI with total energy expenditure when both are expressed as multiple of BMR. In stable weight conditions, the ratio of EI to BMR is equal to the ratio of total energy expenditure to BMR (EI/BMR = total energy expenditure/BMR). The total energy expenditure: BMR ratio is also implied as PAL. Hence, the cut-off values are the 95 % confidence limits of agreement between the EI to BMR ratio and PAL. The BMR in kilocalories per day (kcal/d) was estimated using population-specific BMR equations validated for Malaysian adolescents^(28)^.

BMR equation for male adolescent:
(1)

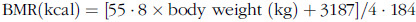




BMR equation for female adolescent:
(2)

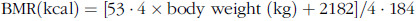




In the Goldberg equation:
(3)

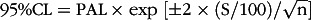

the number (*n*) was equal to one because detection of misreporting was at the individual level. As proposed^(10)^, the age-specific PAL (low PA = 1·6, moderate PA = 1·8 and vigorous PA = 2·0 physical activity) adapted by European Food Safety Authority for individuals of age 15–17 years were assigned to all individuals. The application of individual PAL in the equation is highly recommended since it has been indicated to enhance sensitivity without the loss of specificity^(7)^. While S is the factor that accounts for variation in EI, BMR and energy requirements are given by the equation:
(4)

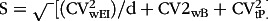

The CV_wEI_ is the within-subject CV in EI; d is the number of recording day; CV_wB_ is the within-subject CV in repeated BMR measurements and CV_tP_ is the total variation in PAL. To calculate S for 30 d of food consumption (d) based on the FFQ, the proposed revised CV was substituted into the equation^(10)^ considering 23 % of CV_wEI_, 8·5 % of CV^2^_wB_ and 15 % of CV_tP_. After substituting the estimated values into the equation, adolescents who fell below the lower limit (EI: BMR < PAL) or above the upper limit (EI: BMR > PAL) of accurate reporting were considered as under-reporters and over-reporters, respectively, whereas adolescents were considered acceptable reporters if their calculated ratio of EI: BMR fell within the two limits.

### Statistics

The assumption of non-normality for each variable including EI for sample sizes greater than 300 was determined based on histograms and skewness values larger than 2^(29)^. All variables were found to be within an acceptable range. The descriptive mean values between dietary reporters were tested for differences with a two-way ANCOVA followed by Bonferroni post hoc after correction for age and gender. Determinants for misreporting were analysed using logistic regression. A univariate or crude model was conducted separately for each of the potential variables. To examine the independent association, a multivariate-adjusted logistic model was created by entering all variables that were found significant in the univariate analysis simultaneously.

The utility of the statistical method when investigating the relationship between BMI and EI was tested with multivariate linear regression. The following models were examined and compared with correct implausible reporters: the first model was adjusted for all covariables (including all participants), the second model included only plausible recalls, the third model was similar to the first model but additionally adjusted for the reporting groups (under-reporter and plausible reporter). The variance inflation factor calculated for each regression model revealed no critical levels of multicollinearity that warrant corrective measures. All statistical analyses were performed using the Statistical Package for Social Science (SPSS) version 24 software (SPSS Inc.). A *P* value <0·05 two-tailed was considered statistically significant.

## Results

Characteristics of the study sample are presented in Table 1. The prevalence of under-reporter was 17·4 %. No adolescents were identified as over-reporters. Male adolescents were twice highly (23·4 %) to under-reporting than female adolescents (11·5 %). Adolescents of Chinese descent and urban areas under-reported more than adolescents of other races and school types. Adolescents who under-report had BMI-*Z* scores, percentage of fat, lean mass and BMR higher than acceptable reporters.

**Table 1:** Participant characteristics across categories of reporting status in Malaysian adolescents (*n* 917)

	Under-reporters (*n* 160, 17·4 %)		Acceptable reporters (*n* 757, 82·6 %)		All (*n* 917)	
	n	%	*n*	%	*n*	%
Gender						
Male	108·0	23·4	353·0	76·6	461·0	50·3
Female	52·0	11·4	404·0	88·6	456·0	49·7
Ethnicity						
Malay	94·0	15·7	506·0	84·3	600·0	65·4
Chinese	47·0	24·9	142·0	75·1	189·0	20·6
Indian	18·0	20·7	69·0	79·3	87·0	9·5
Orang Asli	1·0	2·4	40·0	97·6	41·0	4·5
School type						
Boarding	21·0	15·7	113·0	84·3	134·0	14·6
Urban	86·0	22·3	300·0	77·7	386·0	42·1
Rural	38·0	12·8	258·0	87·2	296·0	32·3
Sport	15·0	14·9	86·0	85·1	101·0	11·0
	Mean	95 % CI	Mean	95 % CI	Mean	95 % CI
BMI-*Z* †	1·4	1·2, 1·7*	−0·2	−0·3, −0·1	0·1	0·0, 0·2
	*n*	%	*n*	%	*n*	%
Under-wt/normal	56·0	8·0	640·0	92·0	696·0	75·9
Over-wt	35·0	28·5	88·0	71·5	123·0	13·4
Obese	69·0	70·4	29·0	29·6	98·0	10·7
	Mean	95 % CI	Mean	95 % CI	Mean	95 % CI
Fat percentage (%)†	26·1	24·2, 27·9*	19·6	18·9, 20·3	20·7	20·0, 21·4
Lean mass (kg)†	53·0	51·4, 54·5*	42·0	41·5, 42·6	43·9	43·3, 44·5
BMR (kcal)†	1594·7	1569·5, 1619·9*	1331·9	1321·1, 1342·8	1378·2	1361·9, 1394·5
PA (MET-m/w)†	1599·0	1437·7, 1760·2	1567·6	1484·8, 1650·5	1573·1	1499·2, 1646·9
	*n*	%	*n*	%	*n*	%
Low	19·0	12·7	131·0	87·3	150·0	16·4
Moderate	103·0	17·5	487·0	82·5	590·0	64·3
High	38·0	21·5	139·0	78·5	177·0	19·3
	Mean	95 % CI	Mean	95 % CI	Mean	95 % CI
SB (h/d)†	8·3	8·0, 8·5	8·4	8·3, 8·5	8·4	8·3, 8·5
	*n*	%	*n*	%	*n*	%
Low	42·0	17·3	201·0	82·7	243·0	26·5
High	118·0	17·5	556·0	82·5	674·0	73·5
Smoking status						
Yes	27·0	25·0	81·0	75·0	108·0	11·8
No	133·0	16·4	676·0	83·6	809·0	88·2
Drinking status						
Yes	15·0	20·8	57·0	79·2	72·0	7·9
No	145·0	17·2	700·0	82·8	845·0	92·1
DS						
Yes	63·0	16·2	325·0	83·8	388·0	42·3
No	97·0	18·3	432·0	81·7	529·0	57·7

wt, weight; kcal, kilocalories per day; BMR, basal metabolic rate; PA, physical activity; SB, sedentary behaviour; DS, dietary supplements; MET-m/w, multiples RMR-min/week; BMI-Z, BMI *Z*-score.

* Significant difference between under-reporters and acceptable-reporters (*P* < 0 05) by two-way ANCOVA adjusted for age and gender.

† Adjusted mean.

Table 2 shows the dietary characteristics for different qualities of reporters. Under-reporters had significantly low dietary intakes compared with plausible reporters for all nutrients except for vitamin C, chromium and fluoride.

**Table 2:** Dietary characteristics across categories of reporting status in Malaysian adolescents (*n* 917)

	Under-reporters (*n* 160, 17·4 %)		Acceptable reporters q(*n* 757, 82·6 %)		All (*n* 917)	
	Mean†	95 % CI	Mean†	95 % CI	Mean	95 % CI
Energy (kcal)	1728·0	1691·0, 1765·0*	2022·3	2006·3, 2038·2	1976·5	1959·5, 1993·5
Macronutrient						
Protein (g)	62·7	61·4, 64·0*	69·8	69·2, 70·3	68·7	68·1, 69·2
Total fat (g)	46·8	45·4, 48·2*	53·2	52·7, 53·9	52·3	51·7, 52·9
Cholesterol (mg)	197·0	189·4, 204·7*	209·0	205·7, 212·3	207·5	204·5, 210·6
SFA (g)	8·8	8·4, 9·3*	10·0	9·8, 10·2	9·9	9·7, 10·0
MUFA (g)	8·5	8·1, 8·9*	9·3	9·2, 9·5	9·2	9·1, 9·4
PUFA (g)	5·7	5·5, 5·9*	6·14	6·0, 6·2	6·1	6·0, 6·2
Carbohydrate (g)	263·9	257·3, 270·5*	316·0	313·1, 318·8	307·9	304·8, 310·9
Total sugar (g)	33·3	31·4, 35·3*	38·6	37·7, 39·4	37·8	37·0, 38·5
Total dietary fibre (g)	3·8	3·6, 4·0*	4·5	4·4, 4·6	4·4	4·3, 4·4
Micronutrient vitamin						
Thiamin (B_1_) (mg)	0·8	0·8, 0·9*	1·0	1·0, 1·0	01·0	0·9, 1·0
Riboflavin (B_2_) (mg)	1·1	1·0, 1·1*	1·3	1·3, 1·3	1·2	1·2, 1·3
Niacin (B_3_) (mg)	10·5	10·2, 10·9*	12·4	12·3, 12·6	12·1	12·0, 12·3
Pantothenic acid (B_5_) (mg)	0·6	0·5, 0·6*	0·8	0·7, 0·8	0·7	0·7, 0·8
Pyridoxine (B_6_) (mg)	0·8	0·7, 0·8*	0·9	0·8, 0·9	0·9	0·9, 0·9
Folate (DFE) (B_9_) (µg)	32·2	30·2, 34·2*	35·7	34·8, 36·6	35·2	34·4, 36·0
Cobalamin (B_12_) (µg)	2·7	2·6, 2·9*	3·0	2·9, 3·0	2·9	2·9, 3·0
Vitamin C (mg)	54·9	51·2, 58·5	61·5	59·9, 63·1	60·3	58·9, 61·8
Vitamin D (µg)	0·2	0·2, 0·3*	0·3	0·3, 0·3	0·3	0·3, 0·3
Vitamin E (mg)	3·1	3·0, 3·2*	3·4	3·3, 3·4	3·3	3·3, 3·4
Vitamin K (µg)	6·2	5·8, 6·6*	6·8	6·7, 7·0	6·7	6·6, 6·9
Micronutrient mineral						
Ca (mg)	431·8	412·0, 451·6*	510·1	501·5, 518·6	496·2	488·2, 504·2
Fe (mg)	14·9	14·4, 15·5*	17·4	17·2, 17·6	17·0	16·8, 17·2
Zn (mg)	4·2	4·0, 4·3*	4·7	4·6, 4·8	4·6	4·6, 4·7
Se (µg)	26·7	25·6, 27·8*	28·9	28·4, 29·4	28·6	28·1, 29·0
Phosphorus (mg)	943·8	921·4, 966·3*	1037·4	1027·7, 1047·0	1022·2	1013·1, 1031·3
Na (mg)	1915·4	1832·1, 1998·6*	2159·7	2123·8, 2195·7	2125·7	2092·3, 2159·1
Potassium (g)	1·2	1·2, 1·2*	1·3	1·3, 1·4	1·3	1·3, 1·3
Mg (mg)	124·3	120·4, 128·3*	135·6	133·8, 137·3	133·9	132·3, 135·5
Cr (µg)	0·9	0·8, 1·0	1·0	1·0, 1·0	1·0	0·94, 1·02
Cu (µg)	545·1	524·6, 565·6*	594·0	585·2, 602·9	587·0	578·8, 595·3
Mn (mg)	0·2	0·1, 0·2*	0·2	0·2, 0·2	0·2	0·2, 0·2
Mo (µg)	1·6	1·3, 1·8*	1·9	1·8, 2·0	1·9	1·8, 1·9
Fl (mg)	0·2	0·2, 0·2	0·2	0·2, 0·2	0·2	0·2, 0·2

kcal, kilocalories per day.

* Significant difference between under-reporters and acceptable-reporters (*P* < 0 05) by two-way ANCOVA adjusted for age and gender.

† Adjusted mean.

Table 3 shows the univariate and multivariate logistics regression model for determinants associated with under-reporting of EI. After significant variables in the univariate analysis were entered and adjusted in the multivariate analysis, the adjusted model demonstrated that adolescents with overweight had 5·5 times higher odds to under-report, while adolescents with obesity had thirty-nine times higher odds to under-report than under-weight/normal adolescents (*P* < 0·001).

**Table 3:** Factors associated with under-reporting in Malaysian adolescents (*n* 917)

	Under-Rep (*n*)/Accept Rep (*n*)	Univariate			Adjusted-multivariate		
		OR	95 % CI	*P*-value	OR	95 % CI	*P*-value
Age (year)		1·0	0·7, 1·3	0·921	–	–	–
Gender							
Male	108·0/353·0	2·4	1·7, 3·4	<0·001	3·2	2·0, 5·0	<0·001
Female	52·0/404·0	1·0	Ref		1·0	Ref	
Ethnicity				<0·001			
Malay	94·0/506·0	1·0	Ref		1·0	Ref	
Chinese	47·0/142·0	1·8	1·2, 2·7	0·004	2·4	1·4, 4·3	0·002
Indian	18·0/69·0	1·4	0·8, 2·5	0·238	1·9	0·9, 4·2	0·094
Orang Asli	1·0/40·0	0·1	0·0, 1·0	0·049	0·4	0·1, 3·0	0·356
School type				0·010			
Boarding	21·0/113·0	1·3	0·7, 2·3	0·430	1·0	0·4, 2·1	0·919
Urban	86·0/300·0	2·0	1·3, 3·0	0·002	1·4	0·8, 2·5	0·316
Sport	15·0/86·0	1·2	0·6, 2·3	0·608	1·7	0·8, 3·8	0·164
Rural	38·0/258·0	1·0	Ref		1·0	Ref	
BMI status				<0·001			
Under-wt/normal	56·0/640·0	1·0	Ref		1·0	Ref	
Over-wt	35·0/88·0	4·6	2·8, 7·3	<0·001	5·5	3·3, 9·2	<0·001
Obese	69·0/29·0	27·2	16·3, 45·4	<0·001	39·1	21·8, 70·2	<0·001
PA level				0·107			
Low	19·0/131·0	1·0	Ref		–	–	–
Moderate	103·0/487·0	1·5	0·9, 2·5		–	–	–
High	38·0/139·0	0·2	1·0, 3·4		–	–	–
SB level							
Low	42·0/201·0	1·0	Ref		–	–	–
High	118·0/556·0	1·0	0·7, 1·5	0·937	–	–	–
Smoking status							
Yes	27·0/81·0	1·6	0·8, 3·1	0·185	–	–	–
No	133·0/676·0	1·0	Ref		–	–	–
Alcohol status							
Yes	15·0/57·0	1·3	0·7, 2·3	0·430	–	–	–
No	145·0/700·0	1·0	Ref		–	–	–
DS							
Yes	63·0/325·0	1·0	Ref		–	–	–
No	97·0/432·0	1·2	0·8, 1·6	0·407	–	–	–

Under-Rep, under-reporter; Accept Rep, acceptable reporter; wt, weight; PA, physical activity; SB, sedentary behavior; DS, dietary supplements; Ref, reference.

Table 4 shows the methods used to correct implausible reporters by examining the relationship between BMI-*Z* and EI. Model 1 which includes total sample found no significant association between the BMI-*Z* score and the reported EI (*β* = -7·66, *P* = 0·089), after the adjustment of demographic variables. After excluding under-reporters, the adjusted multivariate model shows a significant positive association between the BMI-*Z* score and the reported EI (*β* = 36·58, *P* < 0·001). Model 2 contributes to 29·3 % of EI variation (*P* < 0·001). After the additional adjustment for reporting status, model 3 exhibits a significant positive association between the BMI-*Z* score and the reported EI (*β* = 54·50, *P* < 0·001) and contributed to the highest regression coefficient (*R* ^2^ = 0·404, *P* < 0·001) compared with the previous models.

**Table 4:** Impact of under-reporting on the relationship between BMI-*Z* and energy intake (kcal)

	Model 1 (*n* 917)			Model 2 (*n* 757)			Model 3 (*n* 917)		
	*β*	95 % CI	*P*-value	*β*	95 % CI	*P*-value	*β*	95 % CI	*P*-value
Age (year)	−7·7	−36·7, 21·4	0·605	−9·4	−36·28, 17·6	0·496	−3·4	−28·7, 21·9	0·792
Gender									
Male	170·9	140·4, 201·5	<0·001	180·8	152·2, 209·5	<0·001	217·7	190·6, 244·9	<0·001
Female	Ref			Ref			Ref		
Ethnicity									
Malay	Ref			Ref			Ref		
Chinese	−83·9	−128·3, −39·6	<0·001	−79·5	−121·7, −37·2	<0·001	−57·0	−95·8, −18·3	0·004
Indian	−51·8	−109·7, 6·0	0·079	−0·8	−55·2, 53·7	0·979	−15·6	−66·2, 34·9	0·544
Orang Asli	−70·5	−150·0, 9·0	0·082	−99·9	−168·8, −31·1	0·004	−104·1	−173·4, −34·8	0·003
School types									
Urban	Ref			Ref			Ref		
Boarding	90·6	35·2, 146·0	0·001	44·7	−6·2, 95·6	0·085	63·4	15·1, 111·8	0·010
Sport	207·5	150·0, 265·0	<0·001	195·3	141·8, 248·9	<0·001	190·7	140·7, 240·8	<0·001
Rural	84·7	41·1, 128·4	<0·001	67·4	27·4, 107·5	0·001	70·1	32·0, 108·1	<0·001
BMI-*Z*	9·5	−1·5, 20·4	0·089	36·6	24·8, 48·4	<0·001	54·5	43·7, 65·3	<0·001
Report									
Under-rep	–	–	–	–	–	–	Ref		
Accept rep	–	–	–	–	–	–	351·0	310·6, 391·5	<0·001
*R* ^2^	0·213		<0·001	0·292		<0·001	0·404		<0·001

*β*, unstandardised coefficient; R^2^, regression coefficient; Ref, reference; Under-Rep, under-reporter; Accept Rep, acceptable reporter; BMI-*Z*, BMI *Z*-score; Report, reporting status.

Models were tested and compared to correct for misreporting: model 1 was adjusted for all variables (including all participants) (*n* 917).

Model 2 includes only acceptable reporters (*n* 757), model 3 is the same as model 1 but with adjustment of reporting status (*n* 917).

## Discussion

This is the first study to evaluate the prevalence, characteristics and determinants associated with misreporting of EI by applying the Goldberg cut-offs among the ethnically diverse population of Malaysian adolescents. Approximately one-fifth of adolescents were classified as under-reporter, whereas no over-reporter was identified in this cohort. Compared with normal weight, adolescents with overweight and obesity were associated with greater odds of being an under-reporter of EI.

The prevalence of implausible reporters varies widely by study population. On the basis of the Goldberg principles (EI/BMR ratio), the prevalence of under- and over-reporters was, respectively, 26·0 % and 0 % among the French adolescents aged 11–17 years^(13)^, 24·1 % and 2·7 % in a representative sample of US children and adolescents aged 2–19 years ^(4)^ and 4·9 % and 0·4 % among Japanese adolescents aged 15–19 years^(5)^, while the prevalence of under- and over-reporters among the Australian boys and girls aged 14–16 years was 10–11 % (male), 14–15 % (female) and 2·0–2·1 % (male), 14·2–15·3 % (female), respectively^(8)^. In Slovenian adolescents aged 14–16 years, the prevalence of under-reporters was 34 % (male), 27 % (female), while the prevalence of over-reporters was 10 % (male), 11 % (female)^(14)^. High prevalence of under-reporters was observed among French^(13)^, USA^(4)^ and Slovenian adolescents^(14)^, moderate in Australian adolescents^(8)^ while low in Japanese adolescents^(5)^. Except for a study conducted in Australia^(8)^, most of the studies did not measure PAL, thus indicative PAL was assigned to represent average physical activity^(4,5,13,14)^. In particular, study that uses FFQ showed a high prevalence of under-reporters^(14)^; however, other studies that use dietary recall also reported a diverse prevalence of under-reporters ranging from low^(5)^, moderate^(8)^ to high^(4)^.

The variabilities observed in the occurrence of under-reporting across countries may either reflect the actual discrepancy in the accuracy of reporting or disparities of the characteristics of the sample population or the cut-off applied to identify implausible reporters or the dietary assessment instruments or the food composition database. Nevertheless, the current study adds to the accumulating evidence that shows misreporting of EI is a focal issue in dietary surveys, which occurs non-randomly in children and adolescents^(4,5,15)^.

In the current study, implausible reporters were identified using the Goldberg equation and by applying individual PAL. Misreporting in the adolescent population was predominantly under-reporting. Previously, a study among female adolescents has shown that self-reported EI differs approximately 17 % less than the actual intake as estimated using doubly labelled water^(30)^. The current study also noted a moderate percentage of under-reporters (17·4 %). This result describes the acceptable estimation of EI (and possibly food and nutrient intakes) in this cohort. Among the factors that contribute to the high plausible reporters is the application of household-based dietary assessment and the application of wide confidence limits.

Several studies had indicated that under-reporting was more prevalent in female adolescents than in male adolescents^(6,8,16)^. However, equal prevalence of misreporting between boys and girls has also been observed in some studies^(4,5,13)^. On the contrary, in the present study, under-reports were more pronounced among adolescent boys (23·4 % among boys and 11·5 % among girls). The application of age- and sex-specific cut-offs to identify implausible EIs could justify the higher percentage of under-report in boys^(14)^. Boys have higher total energy expenditure compared with girls and thus require higher EI. On the contrary, by applying single EI:BMR cut-off for both sexes, more boys fall above the cut-off and consequently more girls would be identified as under-reporters^(9)^.

While misreporting of EI was common among older children and adolescents, most national studies disregard examining implausible reporters among this age range. The tendency to under-report food intakes among this age group has also been consistently observed in several previous studies^(4–6,8,13,14,16)^. The higher possibility to under-report implies that dietary assessment in this age group is exceptionally challenging. Some of the reasons why this particular age group was more susceptible to under-report were probably due to the extra demands exerted on reporting by increased energy requirements that lead to a more significant amount of food to recall, unstructured habits of eating, substantial numbers of dining out and concerns with self-image^(31)^. Aligning with previous studies, under-reporting of EI affects the estimation of other nutrient intakes in which the macro- and micro-nutrient intakes were significantly lower in under-reporters than acceptable reporters^(32)^. Other studies have also observed selective misreporting of socially desirable food, including high fibre intake among under-reporters^(3,6)^.

A list of potential correlates was examined to identify lifestyle determinants of under-reporting in a multi-ethnic population. The outcome was consistent with the previous studies in which overweight and obese subjects were more likely to under-estimate their food/nutrient intake^(4–6,13,16)^. Under-reporting may indicate the low ability of self-reporting of nutrient intake and denial in this age group (under-recording), but may also reflect real under-eating in an attempt to lose weight. However, body weight must be monitored to assess the degree of under-eating, which is best assessed in a longitudinal study^(3)^. For other determinants such as physical activity or sedentary, research in adolescents was limited to only one study that found under-reporting was significantly associated with increased physical activity and low screen time^(16)^. This may be attributed to increased energy requirements or inaccurate reporting of frequency of eating or inaccurate reporting of portion sizes of substantial quantities of foods^(8)^.

The prevalence of such systematic differentials in this age group accentuates the significant need to correct for reporting errors. Thus far, only a limited number of studies have investigated/explored different statistical approaches to deal with implausible recalls among adolescents. In the first model, no significant association was observed between EI and BMI-*Z*, this result was in line with previous studies^(3,8)^. Conversely, among the acceptable reporters only (as identified in the second model), assessment on the association between EI and BMI-z exhibits that the exclusion of under-reporters led to the establishment of a significant positive association between EI and BMI-*Z*. Even though method 2 can strengthen the diet–disease relationships, it is clear that the exclusion of inaccurate reporters is not an appropriate approach and could result in selection bias^(3,15,33)^.

The regression model with the highest regression coefficient (*R* ^2^ = 0·4) was displayed in model 3, after adjustment of reporting status. The overall model explained 40 % variance (model 3) compared with only 29 % of the variability of the data (model 2) when only acceptable reporters were comprised in the model. Under-reporting in the current study was found to influence the association between reported EI and BMI-*Z*, which emphasises the necessity to contemplate the impact of inaccurate reporters on the data’s validity. These findings corroborated the earlier study in a mixed population of adolescents and adults^(3)^. Despite the limitation of each method, researchers suggested that the best method to recover an association between BMI and EI is to create a dummy variable for the reporting group. Moreover, adjusting for the reporting group would provide a maximum sample size. On the other hand, removing a substantial number of subjects from the analyses would lead to loss of statistical power, and the outcomes can no longer be generalisable to the entire population.

Among the strengths of the current study is the assessment of PAL. Adolescents were assigned according to their individual PALs to increase the sensitivity of Goldberg cut-off method. Yet, the favourable assessment should be of objective measurement such as accelerometer. Still, with the absence of objective measurement, valid estimates of physical activity via questionnaire are better than no indication of specific physical activity categories. The current findings might be specific to this dietary evaluation and should be cautiously interpreted in this notion. The use of a shorter period of dietary assessment may contribute to the low level of misreporting^(34)^. However, it is known that several days of dietary assessment is preferred in providing precise estimates of usual dietary intake and reducing the within-person variability^(35)^. It is worthwhile noting that the substitution of the number of days (d) equal to 30 in the current study was contradicted with the previous study that also used FFQ to measure habitual dietary intake, d was taken as infinite and CV_wEI_ disappeared^(14)^. Nonetheless, this discrepancy is of no real consequence since the prevalence for the two approaches demonstrated a similar value, given that the differences in cut-off between the two approaches for all levels of physical activity were minor.

Misreporting that is linked with overweight and obesity (differential misreporting) should not be entirely precluded because removing subjects with particular traits could lead to selection bias. Likewise, a data set that included misreporting was found to attenuate the association between energy and BMI by increasing the risk of type 2 error. On the other hand, correction for misreporting strengthens the relationship, increases statistical power and better represents the population. Uncovering the problems associated with dietary assessments due to errors in estimation of EI does not suggest that dietary survey should be disregarded because the study of nutrition cannot be secluded from the reality of food intake^(9)^. The Goldberg cut-offs have demonstrated good accuracy in distinguishing inaccurate reports of EI, but misclassifications of under- or over-reporters may still have occurred^(36)^. However, in the absence of objective measures, the Goldberg cut-off is the preferred alternative to mitigate, at least in part, for the problem of energy misreporting. Moreover, the causality of the association presented in the current study cannot be ascertained owing to the cross-sectional nature of the study. To amend this issue, future studies should consider monitoring body weight longitudinally.

## Conclusions

The present study demonstrated the presence of under-reporting in a dietary survey among various demographics of adolescents in Malaysia. However, over-reporting was not evident in this survey. Adolescents with overweight and obesity were more likely to under-report their food intake and consequently affect the estimates of nutrient intakes. As inaccurate energy reporting is the inevitable consequence of differential misreporting, future analyses (based on this data) should consider correcting for implausible reporters so that the influence of misreporting of EI on study outcomes can be acknowledged and consequently improve the precision and accuracy of results from dietary surveys.
